# Changing Home: Experiences of the Indigenous when Receiving Care in Hospital

**DOI:** 10.17533/udea.iee.v38n3e08

**Published:** 2020-11-11

**Authors:** Juan Guillermo Rojas, Raquel Herrero Hahn

**Affiliations:** 1 Nurse, Ph,D. Full Professor, Facultad de Enfermería de la Universidad de Antioquia. Medellín, Colombia. Email: guillermo.rojas@udea.edu.co Universidad de Antioquia Universidad de Antioquia Medellín Colombia guillermo.rojas@udea.edu.co; 2 Nurse, Masters. Professor at Departamento de Enfermería, de la Universidad de Granada. Granada, España. Email: raquelherrero@ugr.es Universidad de Granada Universidad de Granada Granada Spain raquelherrero@ugr.es

**Keywords:** health of indigenous peoples, nursing care, transcultural nursing, hospitalization, anthropology, cultural, salud de poblaciones indígenas, atención de enfermería, enfermería antropología cultural transcultural, hospitalización, antropología cultural, saúde de populacões indígenas, cuidados de enfermagem, enfermagem transcultural, hospitalização, antropologia cultural

## Abstract

**Objective.:**

To understand the meaning of the experience of the indigenous when receiving care in a low-complexity hospital.

**Methods.:**

Qualitative study with ethnographic approach conducted in a hospital of Antioquia, Colombia. The study had 12 indigenous participants who underwent semi-structured interviews. Observation was carried out in hospitalization wards, emergency, and outpatient services of the institution during 40 hours. The analysis process was performed descriptively. The methodological rigor was maintained by applying criteria of confirmability, credibility, transferability, and consistency. The study was approved by the Ethics Committee and authorized by the indigenous authorities to enter the field.

**Results.:**

Five themes emerged: the context of caring for the indigenous, the need to consult the hospital, changes experienced by the indigenous in the hospital, experiences in relation with treatments, and relations established within the hospital. The meaning is constructed from a dichotomous perspective based on the favorable or unfavorable aspects of the situations and experiences, which for the indigenous is like “changing home”.

**Conclusion.:**

The meaning of the experience of receiving care in hospital for the indigenous is constructed from the context in which they live and receive health services, the changes they live in the dimension of space by virtue of their traveling from their vital space to another space that, due to their physical characteristics, results strange and different, even not healing. Upon the difficulties, the indigenous develop strategies and actions to overcome limitations, whether through adaptation and learning.

## Resumen

**Objetivo.:**

Comprender el significado de la experiencia de los indígenas al recibir atención en un hospital de baja complejidad.

**Métodos.:**

Estudio cualitativo con enfoque etnográfico desarrollado en un hospital de Antioquia, Colombia. Participaron doce indígenas a quienes se les realizaron entrevistas semiestructuradas. Se hizo observación en salas de hospitalización, urgencias y servicios ambulatorios de la institución durante 40 horas. El proceso de análisis se hizo de manera descriptiva. El rigor metodológico se mantuvo con la aplicación de los criterios de confirmabilidad, credibilidad, transferibilidad y consistencia. Se contó con aval del Comité de Ética de Investigación de la Facultad de Enfermería de la Universidad de Antioquia y autorización de las autoridades indígenas para entrar al campo.

**Resultados.:**

Emergieron cinco temas: el contexto de la atención de los indígenas, la necesidad de consultar al hospital, los cambios experimentados por los indígenas en el hospital, las experiencias en relación con los tratamientos y las relaciones establecidas dentro del hospital. El significado se construye desde una perspectiva dicotómica con base en los aspectos favorables o desfavorables de las situaciones y experiencias, que para los indígenas es como “cambiar de casa”.

**Conclusión.:**

Para los indígenas, el significado de la experiencia de recibir atención en el hospital se construye a partir del contexto en el que viven y reciben los servicios de salud, los cambios que viven en la dimensión del espacio en virtud de que transitan desde su espacio vital a otro que, por sus características físicas, resulta extraño y diferente, incluso no curativo. Ante las dificultades, los indígenas desarrollan estrategias y acciones para superar las limitaciones, bien sea mediante la adaptación y el aprendizaje.

## Resumo

**Objetivo.:**

Compreender o significado da experiência indígena ao receber atendimento em um hospital de baixa complexidade.

**Métodos.:**

Estudo qualitativo com abordagem etnográfica desenvolvido em um hospital de Antioquia, Colômbia. Participaram 12 indígenas, nos quais foram realizadas entrevistas semiestruturadas. A observação foi realizada no hospital, no pronto-socorro e serviços ambulatoriais da instituição por 40 horas. O processo de análise foi feito de forma descritiva. O rigor metodológico foi mantido com a aplicação dos critérios de confirmabilidade, credibilidade, transferibilidade e consistência. Teve o aval do Comitê de Ética e autorização das autoridades indígenas para o ingresso em campo.

**Resultados.:**

Emergiram cinco temas: o contexto do cuidado aos indígenas, a necessidade de consulta hospitalar, as mudanças vivenciadas pelos indígenas no hospital, as experiências em relação aos tratamentos e as relações estabelecidas no hospital. O sentido é construído a partir de uma perspectiva dicotômica a partir dos aspectos favoráveis ​​ou desfavoráveis ​​das situações e vivências, o que para os indígenas é como “trocar de casa”.

**Conclusão.:**

O sentido da experiência de receber cuidado no hospital para indígenas é construído a partir do contexto em que vivem e recebem os serviços de saúde, das mudanças que vivenciam na dimensão do espaço em virtude da passagem de seu espaço de moradia para outro espaço que, pelas suas características físicas, é estranho e diferente, nem mesmo curativo. Diante das dificuldades, os indígenas desenvolvem estratégias e ações para superar as limitações, seja por meio da adaptação e do aprendizado.

## Introduction

Analysis of the cultural issues immersed in care situations seeks to promote a therapeutic, sensitive, and respectful relation with the cultural diversity from which is derived knowledge that guides the practice, both of nursing professionals, as well as of other professionals involved in caring for people with diverse cultural characteristics. In the intersection of anthropology and a nursing, the cultural issues of care emerge that have been gathered and framed within Transcultural Nursing,(1) creating a field of practical analysis, study, and development that has evolved and has expanded with research and contributions by other theorists, to understand culture as a fundamental element in the interaction among care actors.

Culture is a term with multiple meanings; this study used the theoretical assumptions by Leininger(1) who defines it as a group’s beliefs, values, and lifestyles that are learnt, shared, and transmitted among generations, influencing the ways of thinking and action; on the same way, Purnell(2) adds to the cultural concept the behavioral patterns, lifestyles, artistic creations and thoughts that are socially transmitted and which guide perspectives on the world and decision making by the people. Spector(3) adds the conceptualization on the diversity of traditions in health as part of the cultural heritage that conditions actions, behaviors, and experiences people have about health and disease.

Colombia is a multiethnic and multicultural country, situation determined by the historical coexistence of diverse ethnic groups, mestizo processes, and explicit recognition in the Political Constitution of 1991 of territorial autonomy and protection of ethnic and cultural diversity as pillars of the nation.(4) From the constitutional framework, minority ethnic groups, among them the indigenous, have been object of cultural and social recognition. The indigenous in the country constitute 4.4% of the population,(5) are distributed throughout the territory in nearly 733 indigenous reservations, but principally in dispersed rural zones, border zones, and uninhabited territories, in their majority lacking basic infrastructure and of difficult access.(6) In the Department of Antioquia coexist Embera, Senú, and Gunadule or Kuna Tule indigenous ethnic groups that add up to 37,628 people, distributed in 31 municipalities and organized in 193 communities and 51 reservations. The Embera are the most-numerous ethnic group, by virtue of their inhabiting in 23 municipalities of the department. As macro-ethnicity, among the Embera there are subgroups identified based on eco-cultural adaptations, for example, the Eyabida or mountain people settled in the west and Urabá; the Chamibida in the southeast; and the Dóbida or river people who inhabit in Urabá and the zone of Vigía del Fuerte.(7)

The living and health conditions of the indigenous peoples have been framed by complex dynamics of historical, social, and environmental change linked with the expansion and consolidation of sociodemographic groups in distinct regions and other phenomena linked with rurality, poverty, lack of action by the State and the effects of violence that for years have struck these co-nationals with greater rigor.(6) The epidemiological data of morbidity and general mortality, maternal mortality, neonatal and infant mortality of the indigenous report higher figures than those from the general population, revealing, on one side, socioeconomic barriers of access to basic health services, discrimination due to ethnic reasons, inequity, lack of cultural recognition(8) and, on the other, difficulties related with emerging conflicts due to the cultural characteristics that affect and limit interaction with the health staff.(9) Adding to the aforementioned that research focused on the phenomena of health and ethnicity in Colombia are not studied in depth possibly due to gaps in the epidemiological data and lack of registry and monitoring systems that include the variable of belonging or self-recognition to an ethnic group,(10) with the aggravating condition that people belonging to these groups, like the case of the indigenous, have poor health results.

The socio-sanitary conditions and problems described in the indigenous communities in the country keep similarities with other aboriginal groups around the world. McKonkey(11) found that the indigenous faced economic and social challenges when seeking health services, alluding to geographic and cultural issues; Goodman *et al.,(*12) indicated the growing evidence of inequities in health experienced by Canadian aborigines due to racism and systematic discrimination. Hautecoeur *et al.,*(13) described geographic, economic, and cultural barriers that limited the use of hospital services, concluding that these were not adequate or sufficient to respond to the needs of the indigenous, issues ratified by Li(14) who indicated that cultural barriers were decisive when addressing the inequities existing in the health care of the indigenous.

Besides the situations stated, it has been documented that the experience of hospitalization supposes a challenge for the indigenous from feeling disoriented and the lack of consideration for cultural and spiritual aspects (15). Likewise, it has been described that stereotypes of racial stigmatization condition negatively the attitudes and actions of those who provide care,(12) causing non-satisfaction of their needs and for relations with health professionals to be deficient.(15) Upon this panorama, the experience of rejection perceived by the indigenous during care has as consequences disengagement or delay in care,(12) communication problems, and poor health results.(15)

With these considerations, it is needed for health professionals to evaluate the indigenous patients in their cultural context to bridge existing gaps in care,(12) incorporate cultural aspects during hospitalization, and contribute to diminishing the inequities and ethnic and racial disparities(15) to improve health responses (14) and provide holistic and quality care.(16) Under the assumption that the cultural characteristics of the people condition their health experiences, the present study was developed by starting with the question: How is the experience of the indigenous when receiving care in hospital? A question that gains relevance in the current moment in which the health system transformations have incorporated elements for the adoption of territorial approaches and harmonization with cultural aspects in caring for the indigenous communities contemplated in the Indigenous System of Intercultural Health,(17) an aspect in which Colombia and other countries have advanced in regulating the application of traditional medicine with the purpose of seeking equity in health care, respect for cultural diversity in the commitment to improve the living and health conditions of the indigenous communities.

This text describes and analyzes the findings of the study conducted with indigenous patients in the context of a low-complexity hospital institution in western Antioquia, Colombia to understand the meaning of the experience of receiving care in the hospital for the indigenous, to examine the reality of care and generate contributions to enhance the capacity of the health system to provide sensitive health care congruent with the cultural characteristics of the people.

## Methods

Qualitative research with ethnographic approach was conducted, in external consultation areas and outpatient services, hospitalization ward, emergency, and nutritional recovery ward of a low level of complexity public institution, located in western Antioquia, Colombia. This methodological approach was useful to study an indigenous group as people with determined cultural traits to describe and comprehend reflexively the language, behaviors, and practices in the hospital context.(18) The study had participation from 12 indigenous Emberá from the Eyabida ethnic group, who were selected through purposeful sampling. Of the participants, five were patients, three were companions, and four corresponded to key informants who, due to their role as indigenous leaders, provided testimonies that permitted broadening the description and analysis on the social and cultural situation of the indigenous. Initially, an approach was made with the Indigenous Governor to secure authorization as traditional authority and, through him, contact was established with the rest of the key informants. The other participants were contacted in the hospital, while in condition of patients, companions in hospitalization wards and emergency, or users of outpatient services.

To establish communication, the study was aided by an indigenous translator who spoke Spanish and who was recommended by the Indigenous Governor. Due to his leadership condition, the translator acted as gatekeeper and cultural mediator because he knew the hospital, members of the health staff, the technical language in health, and institutional processes and procedures; the translation task was compensated economically. The participants were contacted directly by the researchers in the hospital facilities (face to face). Participants were explained the objective of the research in their native language and were asked to grant their informed consent. In this regard, only five participants signed the consent and the remaining seven gave their oral consent, given that for the indigenous the spoken word is worth more than the written document. Three indigenous individuals contacted decided not to participate in the research.

Data was collected during 10 months, through 13 semi-structured interviews conducted directly by the team of researchers (a man and a woman experts in research) with support from an interview guide and intermediation by a native Embera translator. The interviews were carried out with the participants only in the hospitalization ward, emergency ward, or in a consultation office, with an approximate duration of 40 minutes, during which were propitiated privacy conditions for the conversation. The testimonies were recorded in digital files for their subsequent transcription, reading, and analysis. The observations made focused on the context, interactions, and communication with the indigenous, and among these with the members of the health staff and other people within the institution. A field diary contained the notes and registries of observations during the field work. The study used 40 hours of observations made in the hospitalization ward (24 hours), emergency (12 hours), and external consultation (12 hours) at different hours of the day, especially during the mornings because it was the period of higher attendance of the indigenous to hospital services.

The transcriptions of the interviews and the field diaries were subjected to the ethnographic descriptive analysis process, performed manually by the researchers. The texts were read line by line looking for units of meaning among which codes were identified. By using analytic files, the codes were grouped to construct the categories and organization of the themes. The analysis made parallel to the data collection permitted developing the theoretical sampling to broaden the inquiry on the phenomenon. The analysis concluded when data saturation was reached in the categories. With the emerging categories, a map was elaborated that served to establish the logical connections among them and facilitate the theoretical involvement in the final stage of the analysis.

The study followed rigor criteria based on strategies of confirmability, credibility, transferability, and consistency; (19) this was supported by an Embera indigenous translator and the interviews and field diaries were transcribed directly by the researchers in the earliest possible time. Reflexivity played an important role throughout the research process; nevertheless, it gained special worth during data collection and analysis in which the researchers as instruments were immersed within the phenomenon to explore it in depth, remove bias, and reflect on the richness of the testimonies by the participants. The transcriptions were shared with some participants and key informants to establish if the findings described reflected their experiences in the hospital. During this stage, the comments by the indigenous served to broaden the dense description of the context and experiences. Finally, to ensure the consistency of the findings, triangulation processes were conducted of techniques when contrasting testimonies from interviews with notes from the field diaries; by researchers through revision and discussion of the findings with the members of the research group and contrast with that reported in the literature.

The study was authorized by the Indigenous Organization of Antioquia, as social entity representing and defending the rights and claims of the indigenous peoples; in this same sense, authorization was also obtained from the hospital’s management to carry out the field work. In compliance with ethical requirements, endorsement was obtained from the Research Ethics committee of the Faculty of Nursing at Universidad de Antioquia and authorization by the Highest Indigenous Governor in the region, as maximum authority representing the participants. Commitments acquired were respected with the ethical endorsement, among them the informed consent. Only five participants signed the consent and the remaining seven granted oral consent, considering that for the indigenous the spoken word is worth more than the written document. This situation was corrected by having authorization from the local indigenous authorities. Confidentiality of the participants was respected, which is why each interview was identified with an alpha-numerical code and data alluding to places, the institution, and the people were omitted.

## Results

Twelve indigenous participated, four were key informants of male gender who acted as indigenous leaders. Of the rest of participants, seven were women and one man. Ages ranged between 21 and 48 years. Women participants were dedicated to household chores and to teaching in primary school, while the men worked as teachers or day workers (farmers). The reports by the participants alluded to their recent or past experiences as patients or companions, principally of children hospitalized and users of the institution’s outpatient services. In the analysis process, five themes emerged that configure the experience of the indigenous: the context and conditioning on health care, the need to consult the hospital, changes experienced in the hospital, experiences regarding treatments, and relations established within the hospital.

### The context of caring for the indigenous

The study was conducted in a hospital to the west of the department of Antioquia, which offers low-complexity services. These types of institutions have the physical infrastructure, technological capacity, and human resource to care for and solve non-complex health problems and carry out activities to promote health and prevent disease with support from a team of professionals in areas of medicine, nursing, bacteriology, dentistry, nutrition, pharmaceutical chemistry, and basic health. In this scenario, care is provided by following institutional norms and protocols derived from the current model of providing services and the canons of western medicine. In the testimonies by the participants, the first element that emerges is the *tambo*, which is the habitual place of residence of the indigenous. In it, or in its surroundings, take place vital individual, family, and collective processes to guarantee subsistence, satisfy basic needs for food, rest, health care or congregation to perform rituals and collective ceremonies: *I had two children who were born in the tambo… I don’t know why… custom I guess…* (E4 P1, woman, p3).

The meaning of the experience of care in the hospital is constructed from a dichotomy that reflects the favorable and unfavorable aspects that surround it and which include the decision to consult, travel to the hospital, accreditation of documents, daily experiences within the hospital, treatments, and communication with members of the health staff and other people within the institution. In this sense, the need to travel from the *tambo* to the hospital configures transit marked by various difficulties, for example, abandoning temporarily the activities that guarantee them economic subsistence, travel long distances walking or in scarce transport means available, and the costs of said travel: *I work, in these nine days I have left the job, I… I am a day laborer… yes, in the fields because I have… to work in plantain crops…* (E11 P5, man, p1); *From where we come from it takes an hour by motorcycle, but by car two hours… it’s far and say to (mentions the name of a township), there are people who have to walk two days, so it’s more complicated*. (E7 P4, woman, p3).

Once in the hospital, the indigenous face difficulties, like not finding medical appointments available and being requested *documents* to prove their affiliation to the General System of Social Security in Health. On one side, travelling from remote zones does not permit the indigenous to arrive on time to solicit services, and on the other, they do not always have the identity documents required in the institution to verify the rights and affiliation to the system, because in the cultural tradition of the indigenous, importance is granted to the spoken word and to the name of the person as a custom inherited from the ancestors, which is why they do not find it necessary to be verified through documents. Within this scenario, emerge the obstacles and delays to receive care: *sometimes you get to the hospital apart and if you can’t get the number they say to wait, to wait…[….] because sometimes there is a doctor, there are three doctors, not enough, too many people…* (E9 P6, woman, p3); *…I was first asked for the document and sent me back home, I was told to first get the identity documents to care for my daughter, but she was already in serious condition, they do the same in XXX (mentions the name of a township) also, if you do not have the document you are not provided care…* (E7 P4, woman, p8).

From the difficulties described, the participants describe the strategies that permit their overcoming such, for example, use of the passage house or shelter offered by the Administrative Entity of the Indigenous Benefits Plan, while they manage to solve the health problems and receive effective support from indigenous leaders, who besides serving as translators, are mediators with the directives and personnel, both in the hospital as in other government entities to carry out the necessary procedures and get the documents required for care.

### The need to consult the hospital

Upon situations that affect health, the indigenous turn first to the traditional medicine men or *jaibanás* from their communities as initial resource due to their closeness and availability to care for them. In this meeting, the traditional medicine men make an initial assessment of the sick, which serves to establish the type of illness and the options they have to facilitate the cure (herbs, prayers, beverages). Thus, decide if it is a disease of the indigenous or if it is a problem that must be solved by the doctor in the hospital, case in which, they recommend traveling and seeking institutional care. This decision is decisive for the indigenous to travel or not to the hospital, under the consideration that the traditional medicine man has the capacity to cure that which the doctor from the hospital cannot. Based on this, the traditional medicine man cures problems, like evil eye, malaise, headache, stomach ache, and other conditions, like malaria, leishmaniosis or hepatitis must be seen in the hospital: *Because of that, we first go to the traditional medicine man, when the medicine man says that… that you don’t have a disease of the indigenous, he sends you to see the doctor... diseases, no for, for me, for example is a pain, a discomfort, and thus […] you cure them with plants, plants also exist for that, for pain, for stomach pain, headache, discomfort and so on, and when the medicine man does not find a disease, he then tells you to come here (referring to the hospital)* (E6 P3, woman, p13); *…that she feels well given that during the treatment, that she has felt improvement because later she will be recovered from her disease she has, like leishmaniosis… says that maybe… that she until the moment has not… has not known of any traditional medicine man who cures that disease* (E4 P1, woman, p2).

The severity that covers the health problem is another determining aspect to seek care in hospital without recurring to the traditional medicine man, that is, the indigenous travel directly to the institution when they note that the health condition is deteriorated and complication signals appear, a situation expressed by the participants as “*being screwed*” that is, very severe: *…it is that most of the indigenous I have seen don’t come just like that to the hospital, never, but it has to be very serious that they now can’t have (refers to giving birth)..* (E7, P4, woman, p10); *I had (one) granddaughter who was pregnant, the girl of the house was a mess (in very severe health condition) and I brought her here at night* (E9 P6, woman, p5).

As stated by the participants, the decision to not attend the hospital is conditioned by prior experiences in the institution, so that they sometimes prefer not to consult, given that in spite of dealing with all the difficulties that arise, when they are seen they are prescribed “pills” or “acetaminophen”, which from their view do not solve the health problems, hence, they choose to go directly to drug stores in the location where they are sold the medications that effectively help them to be cured. Thus, they do not go to the hospital and the decision to self-medicate, or be medicated in the drug store compensate the efforts they must make to get up early and travel from faraway places: *…because of that, the indigenous sometimes do not come because they are given acetaminophen, acetaminophen you can buy… imagine you getting up early around here to be here to [….] well. The only thing I say is that the drug… acetaminophen, not that, they say that is the only thing covered by the plan and all those things, but you… you sometimes no, it does not take away, it is to maybe calm you a bit, but that is not to be cured, you have to buy!* (E7 P4, woman, p3), *…then if you are there and are not seen, you come here and buy the drug …here in the drug store …we sometimes go when the plan does not cover the drug, well we have to buy the drug, because only the doctors and when they see you they give you acetaminophen….* (E9 P6, woman, p4).

### Changes experienced in the hospital

Going from the *tambo* to the hospital represents for the indigenous a series of changes in the space in which they live the experience of the disease, a situation expressed as changing home, to allude to the differences they find between the physical characteristics of their habitual place of residence and those of the hospital. The hospital in which this study was conducted keeps a similar pattern to that of other low-complexity health institutions in the department, as far as the type of construction, organization of spaces, organization of care areas, and distribution of the rooms in the hospitalization area; nevertheless, it was possible to observe some adjustments made to care for the indigenous, for example, supplying mats and baskets as part of the furnishings, decorating walls with paintings and engravings allegorical to the healing tables (symbols) and translation of the rights and duties of patients in the Embera language. The evaluation made by the participants of the conditions of the facilities and the resources available in the hospital compared with the physical characteristics of the *tambo* are the basis to establish that the change can have positive and negative effects on the health condition and recovery. In contrast with the positive evaluation, change of the vital space clashes with their expectations because they feel that the hospital is a close and cold place that can make them sicker: *It is better to be sick here in the hospital… here they help you a lot; in the tambo, who goes there to help?, you die … here on the floor of the tambo, well, for me, because I have been here for two years and well for me it seems that around here it is better, there in the tambo there is too much mud, oh no, no!* (E5 P2, woman, p13); *She says that she’s a bit uncomfortable, because the room is closed and cold, then the room needs to be more open, yes…* (E4 P1, woman, p1).

Change in the physical space for the participants changes in the way of satisfying their basic needs, like eating, hygiene, and elimination. In relation to food, the differences the indigenous find between the diet provided in the hospital in comparison with the foods they consume daily suppose a difficulty that implies their consuming food with disgust, not eating or, even abandoning the institution due to this cause: *…the indigenous who live in the forest and all those things eat fish, they eat meat from the forest not too many chemicals, instead what is meat, the meat is bought and mostly has lots of chemicals, right?, then, that’s it …they don’t like it, they don’t like to eat rice much, there are very few who eat rice and all those things …and salad, given that when they come here they get tired a lot and get very bored, that is what happens here when they arrive …*(E7P4, woman, p3).

Satisfaction of hygiene and elimination needs are also subjected to a series of changes that represent difficulties to them, given that they are not familiarized with existing sanitary facilities in the hospital. In the daily lives of the indigenous bodily bathing or evacuation is done in nearby rivers, hence, they do not recognize or know how to use the shower or the toilet; upon this situation, during the hospital stay they take care of their physiological needs in unsuitable places or abstain from doing so. Regarding the lack of knowledge or inadequate use of the sanitary facilities, the participants report that the difficulty is more notorious when scolded and recriminated by the people caring for them due to their “*lack of cleanliness*”. *…and above all that you have to take a shower, that one thing, that the bathroom, imagine that, the indigenous do not the bathroom, as well as farmers, it has to be the brook …* (E6 P3, woman, p15-16); *…sometimes people come like, as they say, we know nothing indigenous, then it’s clear, the part you say, the hygiene, all that is very different, then they tend to treat you badly verbally with those types of situations* (E2 IC2, man, p3).

In spite of the changes and difficulties described, the indigenous develop strategies that permit them to overcome the problems; hence, they adapt to the hospital conditions through a learning process: *…has learned to use it …yes (referring to the use of sanitary services).* E4 P1, woman, p5); *…let’s say, because many times ah is that in the hospital you are not well cared for, you have to adapt to improve the health…* (E6 P3, woman, p12).

### Experiences in relation to treatments

Once in the institution, the treatments received and procedures performed condition the experience of the indigenous because they establish a connection between the time and achievement of a cure reflected on improving the health conditions or disappearance of signs and symptoms. In situations in which, in spite of the passage of time, the participants do not observe improvement in health conditions, they decide to abandon the institution and seek care from the traditional medicine man as option to deal with the problems: *…yes, yes… they cure you well … long time, yes… that is, like eight days, one month, like that* (E5 P2, woman, p4); *As long as he is better, it does not matter, if it is waiting, but does not improve then no, if this doctor does not help to improve we have to take him to another doctor who does help, the traditional medicine man* (E11 P5, man, p4).

Besides the medications as therapeutic strategy, the testimonies by the participants describe procedures that use medical equipment and technological devices unknown to them regarding their use and utility. Intensive use equipment under conditions of severity, recognition of apparatus as sources of cold and, hence, the cause of more illness, are generators of emotional reactions, like the fear and uncertainty that condition the experience: *well, they placed in the child many things, I, I, I, that is, I have never seen him like that, they placed a needle throughout his whole body and had around here, there a thing there that sounded, and now…[…] that is they place on the child … they placed, I don’t know what it is called, they put in his mouth around here, as… I think so, they gave him food through there …I don’t know, given that I have never seen something like that apparatus ….but a tube there like blue there, blue something like that, I didn’t even ask because it was, I was very scared because … because it was like, oh no!, more horrible there! [….] no, I would get like the child was like sleeping and they put that thing through the stomach, then I thought well with that thing maybe he … that is it made everything cold …then the doctor told me no, the doctor told me not to get frightened, to relax, now, it went well …* (E6 P3, woman, p10-11); *I don’t know apparatus…most of the indigenous, in turn, are afraid of coming to the hospital (*E7 P4, woman, p7).

### Experiences of the indigenous in relations they establish in the hospital

The relations the indigenous establish in the hospital, whether with members of the health staff, personnel from the institution, or other people (indigenous or not), condition the care experience in relation with the communication they manage to establish with the different players. For the participants, all the people who work in the hospital are denominated “*doctors*”, without establishing a distinction among those practicing care work and those who carry out tasks of logistic or administrative support. Independent from this, communication and the difficulties they experience with the people caring for them, are the base for the indigenous to classify people into good or bad. For the indigenous, the “*bad male or female doctors*” are those who do not treat them well, show no interest for their problems, or do not take care of them in timely manner: *…sometimes you get a doctor with the bad doctor who does not like indigenous ….does not understand what …they tell you things in a bad way, ask, because sometimes, like I am saying I, I do say everything, the indigenous we are, sometimes the indigenous come all dirty, sometimes with everything like if they did not comb their hair, with all the clothes that people kind of don’t like, right?, then the doctors get disgusted with the indigenous who come all dirty, they don’t come all cleaned up - rather with all …there are some that one as if they were not people* (E9 P6, woman, p2).

In contrast with that described, other participants value positively relating with the people who care for them in the hospital, given that they manage to establish a better interaction with the people who the catalogue as “*good male and female doctors*”, alluding to their capacity to communicate with them, the good treatment they give them and the interest they display to help them solve problems. As stated by the participants, the communication they establish with the good male and female doctors is supported on the fact that they do not scold or treat them badly, whenever they are in good behavior and are obedient: *…me as an indigenous, I recognize everything, from the doctors here …there are female doctors, there are good female doctors, then they care well, receive you well, ask everything …[….], ah the doctor when he is like very nice with you then they ask what hurts?, since when is it like that?, what do you have?, and you tell them so because they are good …[….] and also when the female doctor is kind of nice does treat you well as women, then you tell her what happens ….you feel confidence, if the doctor is good if you treat him well and he receives you with kindness, yes.* (E9 P6, woman, p2,4,5,6); *…well, I was taken care of well, they treated me well, did not scold, well you have to be obedient and well behaved so you won’t get scolded…[….], but rather, if you are well behaved no, they do not scold you* (E7 P4, woman, p16).

*They do not scold, when you pay attention to them, then they scold you to get the child well organized, or to change the child’s clothes and that* (E8 P5, woman, p3).

In addition to relating and communicating with the members of the health staff, the indigenous communicate with other people in the institution under the same circumstance as patients or companions. Relating is done principally with other indigenous because they feel the need to talk, they do not experience the language barrier and communicate messages that let them understand the situations of the environment to learn and overcome the experience in the hospital.

## Discussion

This study was conducted with the aim of understanding the meaning constructed by the indigenous from the experience of receiving care in hospital. For the participants, assessment of the elements of the context, the need to consult and travel to the hospital, changes experienced on the way to satisfy basic needs, treatments and relations established within the hospital are the aspects that condition the experience described from a dichotomous perspective, represented - on one part - by the difficulties and barriers they face during care, and - on another part - by adapting to the hospital environment, achieving recovery and the learning obtained. The experience of the indigenous of receiving care in hospital is framed within a multicultural moment in which converge two care systems that correspond to different cultural patterns (traditional indigenous medicine and biomedical western medicine), so that a series of situations emerge that configure a series of difficulties among the players, in relation with the ways of understanding living and health processes and how to solve them, configuring the cultural conflict. (20)

The context in which the indigenous live, the activities they carry out in their daily living, their transit from their habitual place of residence to the hospital, and the difficulties they experience to receive health care, comprise a scenario marked principally by adversity, inasmuch as the onset of the disease disrupts their daily lives and, in case of not being able to solve the problems with the resources offered by the traditional medicine man, they must travel to the hospital. Similarly, Arias and de la Cuesta(21) coined the category of unstable equilibrium to describe and analyze the of the internal and external context of the members of an indigenous community in the southeast of Antioquia, which as with the participants in this study, had conditions of insecurity, marginality, poverty, and exclusion, as conditioning factors of the lifestyle and the possibilities of access to health services.

The difficulties expressed by the indigenous configure access barriers to receive care. The first of these is their displacement from faraway places, using scarce transport means available, or even walking long distances. Similar findings were described by Patiño,(9) McKonkey,(11) Goodman *et al.,* (12) and Hautecoeur *et al.,*(13) who reported geographic barriers as an essential element of the inequity the indigenous live in other contexts, so that the conditions of inaccessibility are the principal limitation for the indigenous to use health services. Other difficulties described by the participants in this study allude to not finding medical appointments available or not having the documents required in the institution to provide them care. Maldonado-Sierra(22) documented that the indigenous face difficulties in access to health services due to the lack of identity documents to verify their membership to the health system and the lack of transport means from faraway places. Not having identity documents is one of the principal difficulties the indigenous experience when requesting services from the institution because identification is necessary to access the benefits of the health plan of the subsidized regime; conflict emerges because within the indigenous Cosmo vision the name bears special importance to identify oneself within the community and is part of the way it reaffirms the individual and collective cultural identity, which in terms of Ramírez implies that orality and writing are fundamental elements of the historical traditions of the communities with the purpose of not losing the historical and cultural roots of each community.(23) Seen in another way by the indigenous, excessive administrative procedures cause rejection and limit their possibility for care.(8)

In spite of progress to increase health coverage of the general population and promote membership of the indigenous communities onto the General System of Social Security in Health, insurance is not the only obstacle to overcome, nor is it the guarantee that the indigenous will use health services, inasmuch as barriers persist at cultural, administrative, geographic, and financial levels that limit access.(8) In general terms, as with that described in this study, other authors have emphasized on that the cultural barriers cause inequity and discrimination in health services (11)(14).

Within the context of this study, there is an administrative entity of the health benefits plan for the indigenous communities for the health insurance and care required. Likewise, the hospital has implemented strategies of intercultural approach through dialogue with members of the indigenous community and the physical adjustments necessary for care. However, according to that described by the participants in this study, the adjustments and resources are still insufficient; an aspect similar to that indicated by Ariza(8) that sometimes positive actions (arrangements, adaptations, adjustments) are seen by the indigenous as a sneer and an imposition by the system that still has not generated good results. Upon this, emerges as a point of reflection on how intercultural approach and adaptation strategies come about as a result of the required negotiation processes. For the participants in this study, going to the hospital in search of health care is not the first option. As with other human groups, they try to satisfy health needs with resources available in the community, whether the traditional medicine man, whom they also call *jaibaná* and even *raicero*. The decision to consult the traditional medicine man is supported on the evaluation they make of the problems the indigenous consider are under their capacity to solve. A similar situation was documented by Ariza(8), indicating that traditional medicine is the first therapeutic option due to the cultural roots and the possibility of solving the health problems that western medicine cannot; given that the idea of healing among the Embera is much broader, and the *jaibaná* is in charge of curing the people and the environment, contributes in the construction of collective and political identity (24) and transcend the medical task, approaching that of the priest who has the capacity to manipulate both the spirit and the disease.(25)

Although this research did not propose the objective of revealing the meaning of health for the indigenous, the findings indicate that when they look for care in hospital, they expect to be healed or to have the problem totally solved, based on the conception they have of health and disease founded on the natural balance derived from the construction of relations with spiritual beings, the environment, equilibrium between these, restrictions and prohibitions, and good eating habits (26). For the Embera, the origin of ailments and their classification as diseases caused by God, by whites, or by the malevolence of other *jaibanás*, takes root in the representation they have as existing entities, manipulated by mythical beings of different forms, with diverse intentions and which can be treated or not by the *jaibaná,*(25) or in their moment by western physicians according to the decision made at the time by the traditional medicine man.

Once in the hospital, satisfaction of basic needs and administration of treatments is conducted within a frame of difficulty, given that identity and cultural traits are not valued or taken into account to care for the indigenous in many aspects, among them hygiene and feeding, which is why the cultural conflict is configured (20) because of the cultural differences and divergence in the representations on health and disease between the indigenous and those caring for them. The cultural conflict in question emerges when each player reflects the hereditary consistency rooted in their traditional culture that frames the differences in the beliefs and conducts adopted with respect to health and disease and which have as object to preserve the traditional cultural heritage.(3,27) Within these considerations, for the indigenous the experience of care in health services fulfills, in most cases, a restrictive and imposing function, as expressed by other authors.(8)

The indigenous establish relations with the people they find in the hospital, whether members of the health staff (physicians, nurses, administrative staff, etc.,) or other patients or companions. The base of these relations is supported on the communication that, although sometimes is basic or even adequate, at other times is limited because of the language barrier, a situation that broadens the frame of difficulty in which care takes place. These types of limitations in communication widen cultural gaps, hinder care (11,13,14), satisfaction of needs, and achievement of the healing expectations of the indigenous. As well as that described in this study, other authors have indicated that difficulties in communication configure scenarios in which caring for the indigenous is postponed, being blamed for their health condition, rejected and systematically discriminated,(11) a situation reflected in the lack of adaptation of spaces and care,(8) ratifying that care is framed within the multicultural moment,(20) characterized by lack of interaction and joint and reciprocal work to solve problems.

Given that the experience described, likewise, has a positive connotation, the indigenous recognize the effort made by the members of the health staff to communicate with them and, hence, deploy all the actions necessary to provide them with good care. Thus is configured the intercultural moment in which, as proposed by Siles(20), there is adaptation and adjustment of the context and forms of communication, resulting in greater approach between the indigenous and those caring for them. This intercultural focus emerges as a result of the strategy of differential care developed in the hospital to harmonize the elements of the institutional care model with the traditional indigenous values, resulting in the reciprocal adaptation processes that condition favorably communication and interaction.(28,29)

This transitional process, from multiculturalism to inter-culturalism, implies understanding the meaning of the health-disease process through the vision of the indigenous, establishing the dialogue of wisdom and strengthening the formation of health professionals of indigenous ethnic origin. The importance of inter-culturalism in health lies in the complementarity and reciprocity linked to the inter-subjectivity that emerges in the encounters between players with different cultural baggage.(26) In this manner, institutional action is required, as well as strengthening the individual capacities of the professionals caring for the indigenous population, that is, develop and increase the cultural capacity. According to Purnell(2), cultural capacity is defined as the health professional’s capacity to provide care supported on the cultural characteristics of the people, to promote comprehension of the human experiences in the health-disease process; which according to Leininger(1) implies the implementation of culturally congruent care in which knowledge and sensitive actions of the care providers adjust to the values, beliefs, and lifestyles of the people as axis of health care. In light of these authors, cultural capacity emerges from the intersection of the *emic and etic* perspectives of the players involved in the care phenomena.

In line with the above, cultural capacity supposes the formation for health professionals to recognize that people deserve respect within their framework of cultural reference, as well as to reflect on their cultural identity to know, understand, and respect the cultural characteristics of the receptors of care, interpret the system of traditional meanings on health and generate mechanisms that diminish existing barriers.(3) Upon examining the level of cultural capacity in Medellín, Giraldo and Escobar(28) found that 41% of those surveyed expressed not having received formation on themes of care and cultural diversity, although considered a fundamental aspect during formation. Similarly, Herrero *et al*.,(29) on estimating cultural self-efficacy on a scale from 1 to 5, found that the score reported for caring for the indigenous was 2.72, below the values reported for the Afro-descendants and Mestizos. All these data highlight the urgent need to incorporate cultural aspects of care as a transversal axis in the formation of health professionals, in an attempt to harmonize disciplinary and professional aspects with the population’s care needs, the constitutional framework and the health legislation in effect.

Enactment of the Statutory Health Legislation that establishes and regulates the fundamental right to health,(30) adoption of the Indigenous System of Intercultural Health (SISPI, for the term in Spanish),(17) the Policy of Comprehensive Health Care (PAIS, for the term in Spanish),(31) and the Model of Integral Territorial Action (MAITE, for the term in Spanish)(32) generate the favorable scenario that allows promoting recognition of cultural diversity in health, which supposes a substantive challenge for all the players in the health system and other sectors involved in social development. The SISPI,(17) understood as the policies, norms, principles and resources, institutions and procedures supported on a conception of collective life in which ancestral wisdom serves as guide for the System, in harmony with mother earth and according with the Cosmo vision of each people, seeking articulation, coordination, and complementation with the General System of Social Security in Health, focuses on maximizing achievements in health of the indigenous peoples; supposes a challenge for health institutions, professionals, and the indigenous, given that it demands arduous work of approach on the social response of the health professions to transcend from the physical, technical, and instrumental toward a process of reflection and self-recognition as social and cultural beings, and of respect and recognition toward other social beings with different cultural characteristics.

For health professionals and especially for those in nursing, the challenge consists in enhancing the processes leading to developing and strengthening the cultural capacity and based on it, carrying out necessary actions in the clinical and community settings to generate the best possible conditions to care for the indigenous. The results of this study and of others that have analyzed health care of the indigenous from public health,(33) in Embera (8,21,26) or Wayúu communities,(9,16) ratify the commitment by nursing of incorporating the concepts of trans-culturalism to ensure safe access of the indigenous to health services. According to McKonkey,(11) the strategy of cultural security may contribute to increasing awareness on the access barriers confronted by the indigenous when seeking health services, reason for proposing the creation of health care sessions in dispersed rural zones, among others. In this same line, various authors (8,14-16) indicate the importance of health professionals contributing to improving the situation of the indigenous and diminishing prevalent ethnic and racial disparities through social interventions and holistic care actions that improve the living and health conditions of the indigenous.

It is important to establish that this study was conducted in a health institution with specific characteristics, hence, the findings must be understood within this context in particular, inasmuch as they correspond to the subjective appreciations of the participants in relation with their experiences during care in hospital. The data illustrate how the context of the indigenous and in which care is provided, conditions the construction of the meaning of the experience, situation that could be transformed while the institutional dynamics and interactions among the players also change over time. Moreover, in spite of the linguistic mediation by a native translator, communication could be limited to understand in broad sense the meaning of the testimonies and narrations by the participants. Likewise, it must be specified that the participants belonged to a particular ethnic group whose cultural and linguistic peculiarities do not reflect the entire cosmogony of the members of the Embera nation throughout the Colombian territory.

To conclude, the meaning of the experience of receiving care in hospital for the indigenous is constructed from the context in which they live and receive health services, the changes they live in the dimension of space by virtue of traveling from their vital space to another space that, due to its physical characteristics, results strange and different, even non-healing. In the construction of the meaning, the treatments received and way of satisfying basic needs play a fundamental role, given that they confront different situations not harmonized with their cultural constructions on the way of caring for health problems or performing the activities of basic life. Undoubtedly, the principal conditioning factor of the experience is the communication they establish in the institution, whether with people who provide them care, with other relatives or companions. In this point, the language barrier constitutes an important limitation from which emerge lack of trust, scarce or null interaction, and poor care results. Nevertheless, the indigenous value positively efforts made by those caring for them to communicate with them and adapt the conditions of the environment for said purpose. They acknowledge that, although the institution has carried out actions of cultural approach, progress is still needed in generating better conditions for care. Against experiences they live in hospital, especially the negative ones, the indigenous develop adaptation mechanisms that permit their overcoming difficulties and learning. Finally, the findings described, understood within the legal health framework with intercultural focus, suppose an important challenge for nursing professionals and health professionals in general to strengthen the cultural capacity and advance in developing actions and interventions of negotiation and cultural adaptation that contribute to improving the living and health conditions of the indigenous communities coexisting in the Colombian territory.

This article provides qualitative evidence for health care to minority ethnic groups, like the indigenous, to be accessible and in harmony with their cultural values through negotiation processes and intercultural approach, founded on the principles of Transcultural Nursing.

## References

[1] McFarland M, Wehbe-Alamah B (2018). Leininger´s Transcultural Nursing: Concepts, Theories, Research and Practice.

[2] Purnell L (2014). Guide to Culturally Competent Health Care.

[3] Spector R (2016). Cultural Diversity in Health and Illness.

[4] República de Colombia Constitución Política de Colombia 1991. Gaceta Constitucional Nro. 116 del 20 de julio de 1991.

[5] República de Colombia, Departamento Administrativo Nacional de Estadística DANE, Población Indígena de Colombia (2019). Resultados del Censo Nacional de Población y Vivienda 2018.

[6] República de Colombia, Ministerio de Salud y Protección Social (2016). Perfil de Salud de la Población Indígena, y medición de desigualdades en salud.

[7] Gobernación de Antioquia, Portal Educativo de Antioquia Culturas Indígenas de Antioquia.

[8] Ariza-Montoya J, Hernández-Alvarez M (2008). Equidad de etnia en el acceso a los servicios de salud en Bogotá, Colombia, 2007. Rev. Salud Publica..

[9] Patiño-Londoño S (2016). Guías bilingües: una estrategia para disminuir las barreras culturales en el acceso y la atención en salud de las comunidades wayuu de Maicao, Colombia. Salud Colect..

[10] Agudelo-Suárez A, Martínez-Herrera E, Posada-López A, Rocha-Buelvas A (2016). Ethnicity and health in Colombia: what do self perceived health indicator tell us?. Ethn. Dis..

[11] McConkey S (2017). Indigenous access barriers to health care services in London, Ontario The Engaging for Change Improving Health Services for Indigenous Peoples qualitative study. UWOMJ.

[12] Goodman A, Kim Fleming, Marwick N, Morrison T, Lagimodiere L, Kerr T (2017). “They treated me like crap and I know it was because I was Native”: The healthcare experiences of Aboriginal peoples living in Vancouver's inner city. Soc. Sci. Med1.

[13] Hautecoeur M, Zunzunegui MV, Vissandjee B (2007). Las barreras de acceso a los servicios de salud en la población indígena de Rabinal en Guatemala. Salud Publica Mex..

[14] Jie Li L (2017). Cultural barriers lead to inequitable health care Access for aboriginal Australians and Torres Strait Landers. Chin. Nurs. Rese..

[15] Mbuzi Vainess BN, Fulbrook P, Jessup M (2017). Indigenous cardiac patients’ and relatives’ experiences of hospitalisation: A narrative inquiry. J. Clin. Nurs..

[16] Angarita Navarro AM, Bejarano Beltran MP (2019). Beliefs and practices of culture care in colombian Wayúu pregnant women. Rev. Cienc. Cuidad.

[17] Colombia, Congreso de la República Decreto1953 de 2014. Por el cual se crea un régimen especial con el fin de poner en funcionamiento los Territorios Indígenas respecto de la administración de los sistemas propios de los pueblos indígenas hasta que el Congreso expida la ley de qué trata el artículo 329 de la Constitución Política. Diario Oficial Nro. 49297 del 7 de octubre de 2014.

[18] Boyle J (2003). Estilos de etnografía. In: Morse J. Editor. Asuntos críticos en los métodos de investigación cualitativa.

[19] Noreña-Peña A (2012). Aplicabilidad de los criterios de rigor y éticos en la investigación cualitativa. Aquichán.

[20] Siles J (2010). La naturaleza dialéctica de los procesos de globalización-glocalización y su incidencia en la cultura de los cuidados. Index Enferm..

[21] Arias MM, de la Cuesta C (2004). El caso de los chamibida en Antioquia, Colombia. Index Enferm..

[22] Maldonado-Sierra AA (2016). Perspectivas y retos de la salud indígena en Colombia. Rev. Nova et Vetera.

[23] Ramírez-Velásquez C (2007). Las comunidades indígenas como usuarios de información. Rev. Inv. Bibliotecológica.

[24] Jiménez-Marzo M (2019). Jaibanismo y colonialidad. Los conflictos entre jaibaná en el Resguardo Embera-Chamí de Karmata-Rua. Antioquia, Colombia. Rev. Kavilando.

[25] Arias-Valencia MM, López-Restrepo AD (2014). Potes de la enfermedad entre los embera. Patogenia y cura.

[26] Cardona-Arias J, Rivera-Palomino Y, Carmona-Fonseca J (2015). Expresión de interculturalidad en un pueblo emberá-chamí de Colombia. Rev. Cub. Salud Pública.

[27] Spector R., Muñoz M (2003). Las culturas de la salud.

[28] Giraldo DI, Escobar MP (2010). Competencia cultural de los profesionales de enfermería.

[29] Herrero-Hahn R, Rojas JG, Montoya-Juárez R, García-Caro MP, Hueso-Montoro C (2019). Level of Cultural Self-Efficacy of Colombian Nursing Professionals and Related Factors. J. Transcult. Nurs..

[30] República de Colombia, Congreso de la República Ley 1751 de 2015. Por medio de la cual se regula el Derecho Fundamental a la salud y se dictan otras disposiciones. Diario Oficial Nro. 49427 del 16 de febrero de 2015.

[31] República de Colombia, Ministerio de Salud y Protección Social Resolución 429 de 2016. Por medio de la cual se adopta la Política de Atención Integral en Salud. Diario Oficial Nro. 49794 del 22 de febrero de 2016.

[32] República de Colombia, Ministerio de Salud y Protección Social Resolución 2626 de 2019. Por la cual se modifica la Política de Atención Integral en Salud -PAIS y se adopta el Modelo de Acción Integral Territorial -MAITE-. Diario Oficial Nro. 51092 del 30 de septiembre de 2019.

[33] Orozco-Castillo M., López-Díaz L (2019). Competencia cultural de enfermeras en salud pública con población indígena. Av. Enferm..

